# MiR-377 targets E2F3 and alters the NF-kB signaling pathway through MAP3K7 in malignant melanoma

**DOI:** 10.1186/s12943-015-0338-9

**Published:** 2015-03-26

**Authors:** Liron Zehavi, Hagit Schayek, Jasmine Jacob-Hirsch, Yechezkel Sidi, Raya Leibowitz-Amit, Dror Avni

**Affiliations:** Center for Cancer Research Sheba Medical Center, Tel Hashomer, Israel; Laboratory of Molecular Cell Biology, Center for Cancer Research and Department of Medicine C, Sheba Medical Center, Tel Hashomer, 52621 Israel; Institute of Oncology, Sheba Medical Center, Tel Hashomer, 52621 Israel; Sackler School of Medicine, Tel Aviv University, Tel Aviv, Israel

## Abstract

**Background:**

The incidence of cutaneous malignant melanoma continues to rise, and once the disease metastasizes it is almost inevitably fatal. We recently reported that a large miRNAs cluster on human chromosome 14q32, implicated in many types of cancers, is significantly down-regulated in melanoma. miR-377, one of the miRNAs located within this cluster, was studied here.

**Methods:**

qRT-pCR was used to quantify miR-377 levels in melanoma cell lines and samples. Melanoma cell lines ectopically expressing miR-377 were generated by stable transfection, mRNA expression was assessed using mRNA arrays and protein expression was assessed by Western blot analysis. Potential targets of miR-377 were identified through luciferase reporter assays. Cellular proliferation, migration and soft-agar colony formation were monitored in control and miR-377-expressing cells using cell biology techniques.

**Results:**

miR-377 is expressed in normal melanocytes but not in melanoma cell lines or samples. Its ectopic stable expression in melanoma cell lines decreased their proliferative and migratory capacity and their colony-forming capability. mRNA arrays of melanoma cells over-expressing miR-377 pointed to several down-regulated mRNAs that have putative binding sites for miR-377 in their 3′UTR, of which both E2F3 and MAP3K7 were found to be direct targets of miR-377.

E2F3, a potent transcriptional inducer of cell-cycle progression, was found to be elevated in melanoma cell lines, but decreased following ectopic expression of miR-377. Ectopic miR-377 also led to a decrease in the activity of a reporter plasmid containing three E2F DNA-binding sites linked to a luciferase cDNA sequence, demonstrating that miR-377 down-regulates E2F3-induced transcription.

MAP3K7 (known as TAK1), a serine/threonine kinase along the MAPK signaling pathway, was over-expressed in melanoma but decreased following ectopic expression of miR-377. MAP3K7 is involved in the activation of NF-κB. MiR-377 over-expression led to decreased activity of a reporter plasmid containing two NF-κB DNA-binding sites and to decreased output along the NF-kB signaling pathway.

**Conclusion:**

Our results suggest that miR-377 is an important negative regulator of E2F and MAP3K7/NF-kB signaling pathway in melanoma cells; it is tempting to speculate that its silencing in melanoma promotes the tumorigenic and metastatic potential of the cells through activation of these pathways.

**Electronic supplementary material:**

The online version of this article (doi:10.1186/s12943-015-0338-9) contains supplementary material, which is available to authorized users.

## Introduction

Cutaneous malignant melanoma is by far the most aggressive, therapy-resistant and deadly form of skin cancer, and its incidence is on the rise [1]. The prognosis for melanoma is good when it is diagnosed early and surgically excised, but prognosis drops significantly when regional lymph nodes are involved and metastatic melanoma is unfortunately rarely curable. Although much progress has been made in understanding the molecular events leading to the initiation and progress of melanoma [2,3], the current therapeutic interventions for metastatic melanoma are not sufficient and only little improvement in survival has overall been made [4].

MicroRNAs (miRNAs) are small non-coding RNA molecules that are generated within cells and play a role in post-transcriptional gene regulation. MiRNAs play a role in almost any cellular biological function. Aberrant expression of miRNAs was found in cancerous transformation and progression. Several miRNA profiling studies in melanoma were published so far (reviewed in [5]), but the picture emerging from these works is far from being clear. One of the largest miRNA clusters is located on chromosome 14q32. This chromosomal area is of great developmental importance, exemplified by severe phenotypes associated with altered dosages of the genes within it in mice and humans [6]. The large miRNA cluster within it has been implicated in many types of cancer [7-14].

Previously, we have identified an almost complete silencing of this cluster in melanoma [15], and began to study the individual effects and targets of several miRNAs from this cluster on melanoma cell lines, focusing on miRNAs whose expression was decreased between benign nevi and melanoma. We already showed that two miRNAs from this cluster, miR-376a and miR-367c, which are significantly down-regulated in melanoma, target the insulin-growth-factor-1 receptor and can decrease the malignant phenotype of melanoma cells upon ectopic expression [15]. Our current work focuses on miR-377, another miRNA transcribed from the 14q32 cluster.

## Results

**Figure 1:**
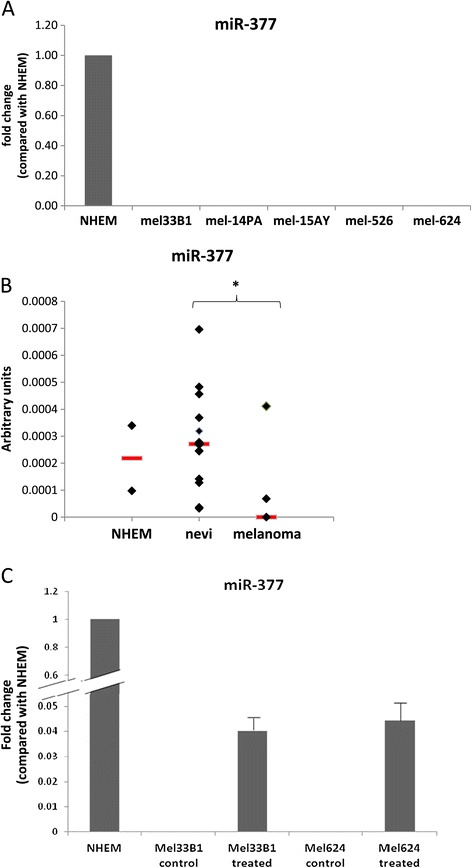
**miR-377 expression in normal melanocytes and melanoma and its re-expression following treatment with epigenetic modifiers. (A)** The expression levels of miR-377 in different melanoma cell lines relative to NHEM was assessed by qRT-PCR and normalized by Rnu48. **(B)** The expression levels of miR-377 in normal melanocytes, thirteen samples of benign nevi and six samples of melanoma as assessed by qRT-PCR and normalized by Rnu43. Each diamond represents one patient. The red thick lines represent the median. *signifies p < 0.05. **(C)** The expression levels of miR-377 in melanoma cells treated with epigenetic modifiers (10 μM 5-Aza and 3 mM PBA) for 96 h, as assessed by qRT-PCR and normalized by Rnu19. The results are presented as relative expression to NHEM. Data is represented as mean ± SEM from three independent experiments.

We previously showed that the large miRNA cluster on chromosome 14q32 is down-regulated in melanoma [15]. Specifically, the expression of miR-377 from this cluster is absent in melanoma cells in comparison to normal human epidermal melanocytes (NHEM) (Figure 1A). Interestingly, in contrast to other 14q32 miRNAs which are already down-regulated at the nevus stage [15], miR-377 is expressed in benign nevi, and its expression decreases in melanoma samples (Figure 1B).

As previously shown by us, treatment of melanoma cell lines with epigenetic modifiers can re-express several miRNAs from this cluster [15,16]. Similarly, miR-377 was also re-expressed when melanoma cells were treated with epigenetic modifiers; whereas miR-377 could not be detected in untreated melanoma cell lines, it was amplified from melanoma cell lines following treatment with a combination of de-methylating agents and histone deacetylase inhibitors (with a threshold cycle of 34–35 in different cell lines), albeit to lower lever than its levels in normal melanocytes (Figure 1C). This result supports the concept that epigenetic modifications take part in the silencing of this miRNA as well.

**Figure 2:**
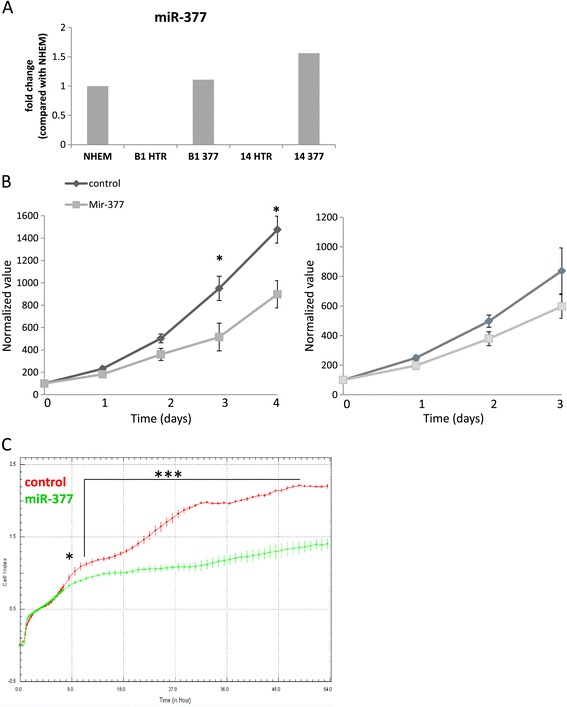
**MiR-377 effect on melanoma cells proliferation. (A)** MiR-377 expression in normal melanocytes (NHEM) or mel33B1 (B1) or mel-14PA (14) melanoma cells after stable transfection with HTR control vector (B1 HTR, 14 HTR) or vector expressing miR-377 (B1 377, 14 377). Rnu48 served as normalizing control. **(B)** The growth of mel-14PA (left) and mel33B1 (right) melanoma cell lines stably transfected with HTR control vector or vector expressing miR-377 was assessed using the crystal-violet method for 72–96 h. Data is represented as mean ± SEM from three independent experiments. *signifies p < 0.05. **(C)** The growth of mel-14PA melanoma cell lines stably transfected with HTR control vector or vector expressing miR-377 was assessed using the xCELLigence real-time system. For determination of statistical significance, a repeated measures two-way ANOVA with Bonferroni post-hoc tests was performed; *signifies p < 0.05, ***signifies p < 0.001.

We generated melanoma cell lines with stable ectopic expression of miR-377 or control RNA, HTR [17] (Figure 2A). Mel-14PA melanoma cells over-expressing miR-377 in a stable manner exhibited a statistically-significant reduction of ~40% in growth relative to HTR-transfected control cells by 96 h (Figure 2B, left panel). This growth pattern was also observed by monitoring cell viability by the real-time cell analyzer (xCELLigence system [18] (Figure 2C). Mel33B1 melanoma cells over-expressing miR-377 in a stable manner exhibited a non-statistically-significant reduction of ~30% in growth relative to HTR-transfected control cells by 96 h (Figure 2B, right panel).

**Figure 3:**
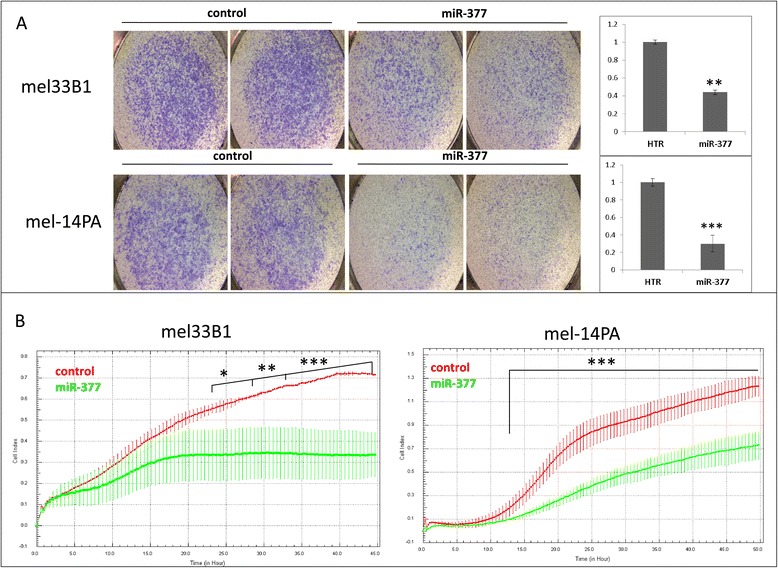
**MiR-377 effect on melanoma cells migration. (A)** The migration of mel33B1 and mel-14PA melanoma cell lines stably transfected with HTR control vector or vector expressing miR-377 was assessed using the in-vitro transwell method 24 h after seeding. Representative micrographs of the transwell membrane are shown; Quantification of the area covered by the cells was performed using the ImageJ software [20] following three independent experiments; p value was calculated by t-test**p < 0.01;***p < 0.001 **(B)** Cellular migration was also assessed using the xCELLigence real-time system (for both cell lines as written above the graphs). For determination of statistical significance, a repeated measures two-way ANOVA with Bonferroni post-hoc tests was performed; *p < 0.05, **p < 0.01, ***p < 0.001.

**Figure 4:**
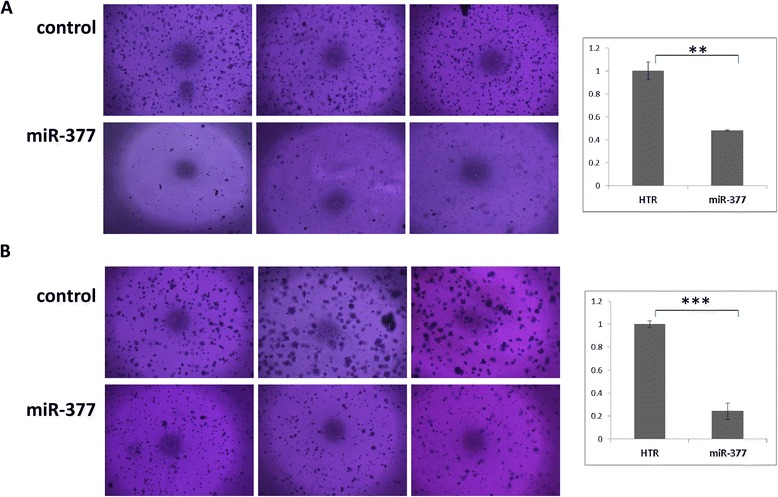
**MiR-377 effect on soft agar colony formation of melanoma cells.** Soft agar colony formation assays were performed using mel-14PA **(A)** or mel33B1 **(B)** melanoma cells over-expressing either miR-377 or HTR control vector. Colonies were stained after 3 weeks in culture. Representative photomicrographs are shown in the left panel. Quantification of the area covered by the colonies was performed using the ImageJ software [20] following three independent experiments (right panel). p value was calculated by t-test; **p < 0.01, ***p < 0.001.

Cellular migration was monitored using an in-vitro transwell system. MiR-377 transfected cells showed significantly decreased migration through a transwell membrane relative to HTR transfected control cells in both mel-14PA and mel33B1 melanoma cell lines (Figure 3A). Migration was also monitored by assessing the number of cells passing through the CIM-Plate of the xCELLigence Real-Time Cell Analyzer system [19]. Whereas HTR-transfected control melanoma cells exhibited a time-dependent migration through the membrane, the miR-377 transfected cells showed significantly reduced migration through the membrane (Figure 3B). Furthermore, over-expression of miR-377 led to a significant reduction in colony formation in both melanoma cell lines (Figure 4).

In order to point out potential mRNA targets of miR-377, mRNA array analysis was performed, comparing miR-377-expressing and control-HTR-expressing mel33B1 and mel-14PA melanoma cell lines. We searched for transcripts whose expression was decreased in the presence of miR-377. The mRNA expression pattern in mel33B1 and mel-14PA cells differs, yet miR-377 has a clear effect on mRNA expression levels in both cells. Using a cut-off of 1.5-fold-change in expression, we found 363 genes whose expression was decreased in miR-377-expressing mel33B1 cells and 110 transcripts whose expression was decreased in miR-377-expressing mel-14PA cells. Ninety seven genes were down regulated in both cells overexpressing miR-377 and 18 were up regulated in both cells over expressing miR-377 (Additional file 1: Figure S1A, and Additional file 2: Table S1). We then used the miRWalk bioinformatics tool (www.umm.uni-heidelberg.de/apps/zmf/mirwalk/index.html) that provides lists of miRNA-predicted targets according to 10 different bioinformatics software. MiRWalk pointed to 2882 predicted targets of miR-377 that appear in at least 4 out of 10 different softwares. When we crossed this list with the list of down regulated mRNAs in miR-377-expressing melanoma cells, we found 4 putative transcripts with miR-377-binding sites: E2F3 (a putative target of miR-377 in 6 out of 10 software), MAP3K7 (in 4 out of 10), KRAS (in 5 out of 10) and CDK6 (in 5 out of 10). The expression level of these 4 mRNAs on the array is provided in Additional file 1: Figure S1B.

**Figure 5:**
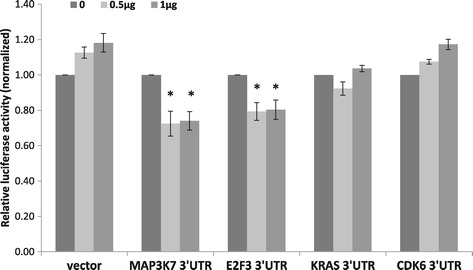
**miR-377 effect on the 3′UTR of selected potential targets.** 293 T cells were co-transfected with control empty psiCHEK-II (vector), or with a vector containing a part of the 3′UTR (containing the putative binding site for miR-377) of the MAP3K7 or E2F3, or KRAS, or CDK6 genes, attached to luciferase cDNA, concomitant with a miR-377 expressing vector at different concentrations (0.5 or 1 μg). The results are presented as the ratio of expression of renilla/luciferase that was normalized relative to control-transfected cells for each vector. Data is represented as mean ± SEM from three independent experiments. *signifies p < 0.02.

In order to find out which of those genes is a true molecular target of miR-377, part of the 3′UTR of each of these genes, containing the putative miR-377 binding site/s, was individually cloned to a psiCHECK-II plasmid downstream to a synthetic Renilla luciferase gene (hRluc). Luciferase activities were significantly inhibited following introduction of miR-377 to cells that were also transfected with luciferase attached to the 3′UTR of MAP3K7 or part of the 3′UTR of E2F3 (Figure 5). This inhibition could not be detected following transfection of luciferase attached to the 3′UTR of KRAS or of CDK6. Hence we decided to focus on the effect of miR-377 on E2F3 and MAP3K7.

**Figure 6:**
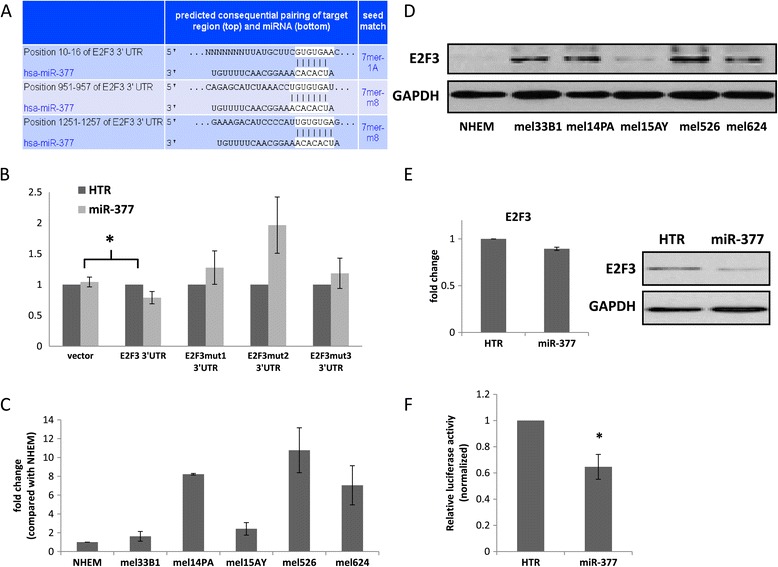
**MiR-377 targets E2F3. (A)** The putative binding sites of miR-377 in the E2F3 3' UTR are shown (taken from TargetScan at http://www.targetscan.org/). **(B)** mel-14PA melanoma cells stably expressing HTR control (dark grey) or expressing miR-377 (light grey) were transfected with psiCHECK-E2F3-WT-3’ UTR-luciferase or psiCHECK- E2F3-mut-3'UTR-luciferase; mutation in site 1 (position 10–16 of the 3′UTR), or mutation in sites 1 + 2 (positions 10–16 and 951–957 of the 3′UTR), or mutation in sites 1 + 2 + 3 (positions 10–16 and 951–957 and 1251–1257 of the 3′UTR) of the E2F3 3′UTR. The results are presented as the ratio of expression of renilla/luciferase that was normalized relative to control-transfected cells for each of the 3′UTR constructs. *signifies p < 0.05. **(C)** E2F3 mRNA levels in different melanoma cell lines relative to NHEM as assessed by qRT-PCR. **(D)** E2F3 protein levels in different melanoma cell lines as assessed by WB. **(E)** E2F3 mRNA (left panel) and protein (right panel) levels in mel-14PA over-expressing HTR control vector or vector expressing miR-377 as assessed by qRT-PCR and WB, respectively. **(F)** mel-14PA melanoma cells stably expressing HTR control or expressing miR-377 were transfected with E2F-BS-luciferase and renilla vector as an internal control. The resulting cell extracts were analyzed by luciferase reporter assay and values normalized to renilla activity. Data is represented as mean ± SEM from three independent experiments. *signifies p < 0.05.

E2F3 has three putative binding sites for miR-377 located in the first half of the gene’s 3′UTR (Figure 6A). The first site is classified as ‘7mer-1A binding’ and the other two sites are classified as ‘7mer-8 m binding’, which theoretically could generate a stronger interaction with the E2F3 3′UTR through an additional nucleotide pairing in position 8 in the “seed” sequence. Targeted mutation of each individual miR-377 binding sites in the 3′UTR of E2F3 abrogated the miR-377-induced decrease in luciferase expression (Figure 6B), confirming that miR-377 directly targets E2F3.

E2F3 mRNA and protein levels were higher in melanoma cell lines compared to NHEM (Figure 6C and D respectively). The effect of miR-377 on the expression of E2F3 mRNA and protein was examined by qRT-PCR and WB analysis of cells over-expressing miR-377. When miR-377 was over-expressed in melanoma cells, the level of E2F3 mRNA was almost unchanged (Figure 6E, left). However, the level of E2F3 protein was decreased by about 70% (Figure 6E, right). This suggests that miR-377 regulates E2F3 expression at the post transcriptional level.

We further asked whether miR-377 affects the biochemical activity of E2F3 using a reporter plasmid containing three E2F3 DNA-binding sites linked to a luciferase cDNA sequence (termed E2F3-BS-luciferase) [21]. Cells over-expressing HTR control vector or vector expressing miR-377 were transfected with E2F3-BS-luciferase vector or empty luciferase vector as an internal control. Over-expression of miR-377 reduced the luciferase activity in the E2F3-BS-luciferase transfected cells, implying that miR-377 led to reduced E2F3 activity (Figure 6F).

**Figure 7:**
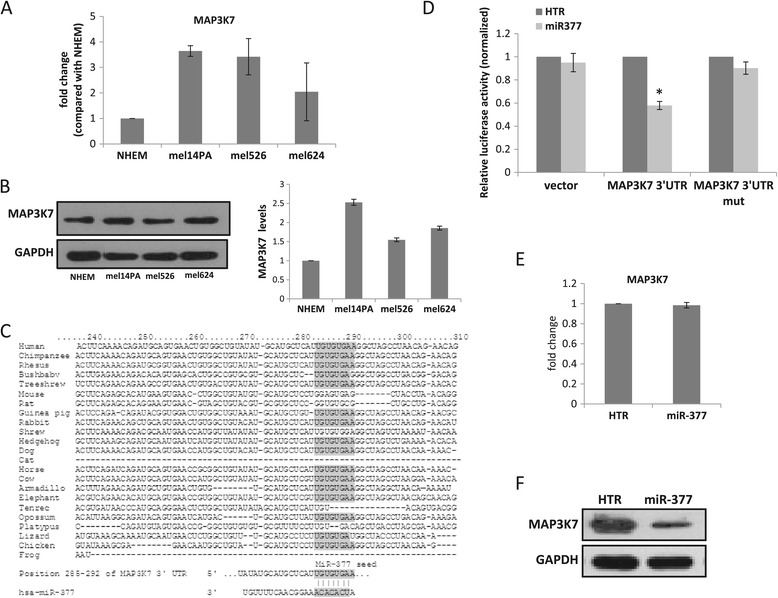
**MiR-377 targets MAP3K7. (A)** MAP3K7 mRNA levels in different melanoma cell lines relative to NHEM as assessed by qRT-PCR. **(B)** MAP3K7 protein levels in different melanoma cell lines as assessed by WB (left). The bar graph denotes the densitometry scanning of the total MAP3K7 bands normalized to the GAPDH bands (right). Data is represented as mean ± SEM of three independent experiments. **(C)** The 3′UTR of the MAP3K7 gene showing potential binding sites for miR-377 (marked), and the sequence conservation from human to lizard in the vicinity of miR-377 seed sequences (taken from TargetScan at http://www.targetscan.org/). **(D)** 293 T cells were co-transfected with a control empty psiCHEK-II-luciferase (vector), or wild type MAP3K7 3′UTR-luciferase plasmid, or mutant MAP3K7 3′UTR-luciferase plasmid, together with a plasmid expressing miR-377. The results are presented as the ratio of expression of renilla/luciferase that was normalized relative to control-transfected cells for each vector. Data is represented as mean ± SEM from three independent experiments. *signifies p < 0.05. **(E)** MAP3K7 mRNA levels in mel-14PA over-expressing HTR control vector or vector expressing miR-377 as assessed by qRT-PCR. **(F)** MAP3K7 protein levels in mel-14PA over-expressing HTR control vector or vector expressing miR-377 as assessed by WB.

MAP3K7 mRNA and protein levels were higher in melanoma cells compared to NHEM (Figure 7A and B, respectively). MAP3K7 has a highly conserved putative binding site for miR-377. This site is classified as ‘8mer binding’ and is located in the second third of the gene’s 3′UTR (Figure 7C). Luciferase activity was significantly inhibited following introduction of miR-377 and the 3′UTR of MAP3K7. Mutation of the MAP3K7 3′UTR binding-site abolished the ability of miR-377 to decrease luciferase expression (Figure 7D), confirming that miR-377 directly targets MAP3K7 3′UTR.

Mir-377 over-expression in melanoma cells did not alter the level of MAP3K7 mRNA (Figure 7E). The level of MAP3K7 protein, however, was significantly decreased by about 60% (Figure 7F), suggesting that miR-377 regulates MAP3K7 at the post transcriptional level.

**Figure 8:**
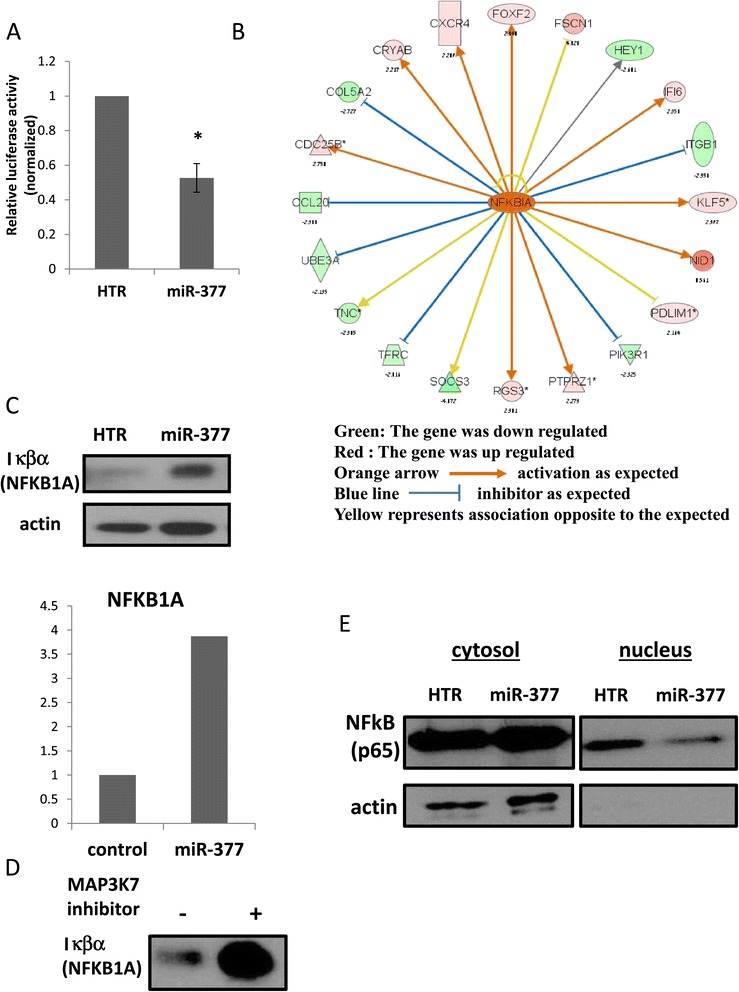
**miR-377 effects on the NF-κB pathway. (A)** mel-14PA melanoma cells stably expressing the HTR control vector or a vector expressing miR-377 were co-transfected with NF-κB luciferase vector and renilla vector as an internal control. The resulting cell extracts were analyzed by luciferase reporter assay and values were normalized to the renilla activity. Data is represented as mean ± SEM from three independent experiments. *signifies p < 0.05. **(B)** Using the Ingenuity Pathways Analysis software suite, we illustrate the network of NFKB1A target genes in melanoma cells over-expressing miR-377. The green and red boxes represent genes that were experimentally found to be down-regulated or up-regulated in mir-377-expressing cells, respectively; the orange and blue lines represent trends of activation or inhibition, respectively, that would be expected following activation of NFKB1A signaling output. The yellow lines represent genes in which a discrepancy exists between the observed change in gene expression and the expected change. **(C)** The protein levels of Iκβα (NFkB1A) were assessed in miR-377 expressing and control melanoma cell by WB and quantified using densitometry with actin levels serving as internal loading control. **(D)** The protein levels of Iκβα (NFkB1A) in mel-14PA melanoma cells after treatment with the commercially available inhibitor 5Z-7-oxozeaenol, 4 mM, for 24h. **(E)** The protein levels of NF-kB in the cytosolic and nuclear cellular fractions were assessed by WB with actin levels (completely absent in the nuclear fraction) serving as internal loading and fractionation control.

MAP3K7 is involved in the activation of NF-κB; MAP3K7 phosphorylates and activates the IKK complex, leading to the phosphorylation, ubiquitination and degradation of NFKB1A (also known as IκBα). This frees NF-κB, which is then activated and trans-located to the nucleus [22]. We therefore hypothesized that over-expression of miR-377 would lead to inhibition of the NF-κB signaling pathway. Using a reporter plasmid containing two NF-κB DNA-binding sites linked to a luciferase cDNA sequence gene [23], we showed that over-expression of miR-377 decreases the reporter luciferase activity by about 50% (Figure 8A), indicating that miR-377 decreases the activity along the NF-κB pathway as well.

Furthermore, functional analysis of the mRNA expression array of cells over-expressing miR-377 showed a fairly consistent correlation between miR-377 expression and regulation of known gene expression signatures of NFKB1A (IκBα) (Figure 8B). This indirectly implies the involvement of miR-377 in the NF-κB pathway as well.

**Figure 9:**
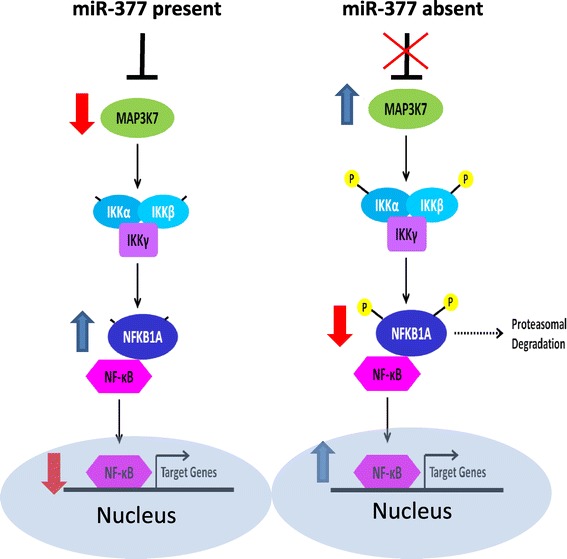
**A model of the effect of miR-377 on the NF-kB signaling pathway.** A schematic model of the potential effect of miR-377 on the NF-kB signaling pathway in melanoma cells. Silencing of miR-377 upregulates MAP3K7. MAP3K7 phosphorylates and activates the IKK complex, leading to the phosphorylation, ubiquitination and degradation of NFKB1A. This frees NF-κB, which is then activated, trans-located to the nucleus and activates transcription of multiple genes.

To verify that the signaling output along the NF-kB is decreased following miR-377 over expression, we assessed the protein levels of NFKB1A (IκBα). NFKB1A levels were higher in miR-377-expressing cells, as would be expected by inhibition of MAP3K7 activity (Figure 8C). Indeed, pharmacological inhibition of MAP3K7 by the commercially available inhibitor 5Z-7-oxozeaenol [24] led to a similar effect on NFKB1A levels as miR-377 over expression (Figure 8D). In addition, the amount of the NF-kB in the nuclear compartment following cellular fractionation was lower in miR-377 expressing cells, suggesting that the translocation of NF-kB to the nucleus is inhibited in the presence of miR-377 (Figure 8E). A schematic model of the effect of miR-377 on the NF-kB signaling pathway is shown in Figure 9.

## Discussion

MiR-377 belongs to a large miRNA cluster on chromosome 14q32 whose expression is silenced in melanoma [15] and in several additional human malignancies including ependymoma [8], osteosarcoma [14], neuroblastoma [11], gastro-intestinal stromal tumors [12], uterine osteosarcoma [9], gliomas [13] and prostate cancer [25]. In fact, this region was already dubbed, several years ago, ‘the largest miRNA tumor suppressor cluster’ [13]. Ectopic expression of miR-377 in melanoma cells significantly decreased their proliferation and migration rates, and led to significant reduction in their ability to form colonies in soft agar, suggesting that miR-377 has tumor suppressor functions as well.

Little is known about miR-377 targets. miR-377 was shown to target FZD4, a gene important for epithelial-to-mesenchymal transition and to affect migration and invasion in prostate cancer cells [25]. A recently published work, in contrast to our current results, showed that miR-377 can target CDK6 [26]. MiR-377 was not shown to target E2F3 until now.

The E2F family of transcription factors controls cell cycle progression. E2F3 is a potent transcriptional inducer of cell-cycle progression and its amplification was strongly associated with invasive tumor phenotype and high tumor grade in a subset of bladder tumors and in prostate cancer [27,28]. It was recently shown that targeting of E2F3 in melanoma cells by miR-203 inhibited cell growth and induced cell cycle arrest and senescence [29]. E2F activity is necessary for the induction of many genes required for S phase progression and Rb inhibits cell cycle progression by repressing the transcriptional activity of E2Fs and their target genes [30]. We found that E2F3, which is over-expressed in melanoma, is a direct target of miR-377 and its activity is regulated by miR-377. E2F1, −2, and -3a proteins are termed “activators” and facilitate cell cycle progression. In contrast, E2F3b has been classified as a repressor E2F, and it negatively controls the cell cycle [31]. E2F3 encodes two protein products; E2F3a and E2F3b, through the use of alternative promoters and different 5′-coding exons. E2F3a and E2F3b share DNA binding, hetero-dimerization and pocket protein binding domains and differ only in their N-termini [31]. Based on the NCBI database, the sequences of the 3′UTR of human E2F3a and E2F3b are the same. The reduction in E2F3 protein levels that was observed following ectopic expression of miR-377 reflects a reduction in the overall levels of both E2F3a and E2F3b, as the antibody used for their detection cannot discriminate between the two isoforms. Indeed, it is likely that miR-377 targets both E2F3a and E2F3b, as their predicted miR-377 binding sites are present in both 3′UTRs. Hurst el al reported that knockdown of both E2F3a and E2F3b in bladder cancer cells induced the most significant anti-proliferative effects compared with knockdown of any one of them alone. Moreover it has been suggested that E2F3b, which is known to negatively control the cell cycle, acts differently when oncogenic stress is present [32].

Additional miRNAs have been shown to down-regulate E2F3. For example, it was recently shown that targeting E2F3 by miR-203 in melanoma cells inhibited cell growth and induced cell cycle arrest and senescence [29]. MiR-449b was found to play a tumor-suppressive role by down-regulating E2F3 expression and reducing the proliferative ability of colon cancer stem cells [33]. MiR-125b was suggested to suppress the development of bladder cancer by targeting E2F3 [34]. In addition, it has been reported that E2F1, 2 and 3 are targets of miR-34a in colon cancer cells [35]. Taken together, these observations point to the importance of E2F3 as a key player in cancer progression. In agreement with these observations, in our initial miRNAs microarray (described in [15]), both miR-125b and miR-34a were down-regulated in melanoma cell lines compared to NHEM; miR-203 and miR-449b were not represented on the array (results not shown).

We found that MAP3K7 is an additional target of miR-377. MAP3K7, also known as TAK1 (TGF-β-activated kinase-1), is a serine/threonine kinase member of the mitogen-activated protein kinase kinase kinase (MAP3K) family, which can be rapidly activated in response to TGF-β signal transduction [36]. MAP3K7 is a key regulator of important cellular pathways associated with the growth of cancer cells (e.g. NF-κB, JNK, and p38 signaling), and was recently shown to have a role in proliferation of melanoma cells [22,37]. Inhibition of MAP3K7 activity induces cancer cell death in pancreatic cancer, suggesting that it may be an effective target for cancer treatment [38]. MAP3K7 regulates both nuclear factor-κB (NF-κB) and mitogen-activated protein kinase (MAPK) signaling pathways that play key roles in development, cell survival, immune response, metabolism, and carcinogenesis. It was recently shown to have a role in proliferation of melanoma cells [37]. Our results corroborate that its levels are elevated in melanoma, and show that miR-377 directly targets it in the post-transcriptional level, leading to a decrease in its protein levels following miR-377 ectopic expression. An additional regulator of MAP3K7 is miR-26b which down-regulates MAP3K7 and inhibits the NF-κB pathway in hepatocellular carcinoma [39]. In agreement, in our initial miRNAs microarray miR-26b was also down-regulated in melanoma cell lines compared to NHEM [15].

Although the precise function of MAP3K7 remains obscure, our work along with previous works characterize it as a tumor promoter in a similar manner to other members of the MAP3K family [40]. Knockdown of MAP3K7 in breast cancer cells inhibited both cell adhesion and invasion and led to reduction of both the incidence and extent of lung metastasis in a mouse model [41]. Furthermore, inhibition of MAP3K7 activity induces cancer cell death in pancreatic and breast cancers [38,42], suggesting that MAP3K7 may be an effective target for cancer treatment. Recent studies also demonstrate that MAP3K7 is associated with lymphoid malignancies [43]. In contrast, other works point to MAP3K7 as a tumor suppressor; deletion of the MAP3K7 gene is significantly associated with high-grade prostate cancer [44] and its loss increased proliferation, migration, and invasion in vitro [45]. Deletion of MAP3K7 in mice liver resulted in carcinogenesis [46]. These reports imply that MAP3K7 can have different roles in different systems and suggest the dual functions of MAP3K7 in tumor initiation, progression, and metastasis as either a tumor promoter or suppressor. The specific role is probably context-dependent and cell type-dependent.

MAP3K7 promotes stabilization and activation of NF-κB; MAP3K7 phosphorylates and activates the IKK complex, leading to the phosphorylation, ubiquitination, and degradation of IκBα, which results in release of inhibition and activation of NF-κB. The freed NF-κB trans-locates into the nucleus and activates transcription of multiple genes involved in pathways controlling immune and inflammatory responses, developmental processes, cellular growth and anti-apoptosis [47]. NF-κB has a critical role in development and progression of many human cancers, including melanoma, in which the expression of NF-κB is up-regulated [48,49]. Suppression of MAP3K7 signaling by dominant negative MAP3K7 reduced NF-kB activation in human head and neck squamous cell carcinoma and breast cancer cell lines [50,51]. In melanoma samples NF-κB pathway is up regulated, affecting survival, proliferation, resistance to apoptosis and metastasis [52]. In fact, it has been demonstrated that up-regulation of NF-κB expression is involved in both the progression of melanoma [53] and the increase in its metastatic potential [54].

We found that over-expression of miR-377 decreased NF-κB-induced luciferase expression by about 50%, significantly increased the expression of the NF-κB-inhibitor NFKB1A, and decreased the nuclear fraction of NF-kB. Our results, taken together, suggest that miR-377 decreases NF-κB signaling pathway output, probably through targeting MAP3K7. The decrease in proliferation, migration and colony formation in melanoma cells over-expressing miR-377 can be a consequence of the down-regulation in MAP3K7 and in the activity of the NF-κB pathway.

MiRNAs have been suggested to act as master regulators of key cellular pathways. Our work shows that miR-377 is a key regulator of several cardinal pathways in melanoma progression: E2F and MAP3K7/NF-κB, rendering it a potential new therapeutic target in melanoma.

## Conclusions

We show here that the expression of miR-377 is completely silenced in melanoma cell lines and samples, and that it directly targets both the transcription factor E2F3 and MAP3K7, a tyrosine kinase along the MAPK pathway, known to be important in the activation of the NF-kB signaling pathway. Ectopic stable over-expression of miR-377 in melanoma cell lines decreased both E2F3 and MAP3K7 protein levels and led to inhibition of proliferation, migration and colony formation. We show that E2F3-induced activity and NF-kB–induced activity are both inhibited in the presence of miR-377, and that miR-377 leads to alterations in the protein levels of both NFKB1A and NF-kB which are in line with decreased output of the NF-kB signaling pathway (Figure 9). It is tempting to speculate that silencing of miR-377 in melanoma unleashes both the E2F3 and NF-kB signaling pathways, promoting the tumorigenic and metastatic potential of the cells.

## Materials and methods

### Cells cultures

Melanoma cell lines were generated directly from metastatic melanoma lesions of patients at the Surgical branch of the NIH (mel526, mel624, mel33B1; [55,56]), or at the ‘Ella institute for melanoma research’ at the Sheba Medical center (mel-14PA, mel15AY) [57]. Their melanocytic origin was verified by qRT-PCR for the melanocyte specific transcript melan-A (Additional file 3: Figure S2). The cell lines were grown in DMEM/RPMI medium supplemented with 10% fetal bovine serum (FBS), 1% Penicillin–Streptomycin (P/S) antibiotics, 1% L-glutamine and 2.5% Hepes solution. All culture medium components were purchased from Biological Industries. Normal human epidermal melanocytes (NHEM, purchased from Promocell) were grown in melanocyte growth medium according to manufacturer’s instructions. NHEM were maintained in culture for up to 5 cycles. All cells were grown in a 5% CO_2_ incubator at 37°C.

### Tumor samples

Formalin-fixed-paraffin-embedded (FFPE) samples of 13 benign nevi or 6 primary cutaneous melanoma were obtained from the pathology institute at the Sheba Medical Center. The initial diagnosis of melanoma and the histological type were verified by a pathologist on the hematoxylin-eosin–stained slides. The tumor or nevus was macro-dissected from the slide in cases in which the sample contained normal tissues as well, based on demarcations delineated by the pathologist. All Nevi were benign, intradermal, some with congenital features. The melanoma samples were of superficial spreading histology (except for one nodular melanoma), 3 of which were ulcerated, with thickness varying from 0.3 to 4.3 mm. The study was approved by the ethics committee of Sheba Medical Center and conducted in adherence to the Declaration of Helsinki protocols.

### RNA extraction

Total RNA was extracted from cell lines using Ambion miRVana™ MiRNA Isolation Kit (Ambion) or Norgen Total RNA Purification Kit (Norgen). Total RNA from 10 sections of 5 μm FFPE tissues was extracted using the Qiagen miRNeasy FFPE kit (Qiagen). Quantity and quality were evaluated using a Nanodrop ND-2000 (Thermo Scientific) with inclusion criteria of A260/A280 ≥ 1.8.

### mRNA micro-array experimentation and analyses

mRNA expression profiling was performed using Affymetrix PrimeView oligonucleotide arrays according to the manufacturer’s protocol. Briefly, 500 ng of Total mRNA was used to generate first-strand cDNA by using a T7-linked oligo (dT) primer. After second-strand synthesis, in vitro transcription was performed with biotinylated UTP and CTP (Affymetrix), resulting in approximately 300-fold amplification of RNA. The target cDNA generated from each sample was processed using an Affymetrix GeneChip Instrument System. Spike controls were added to 15 μg fragmented cRNA before O.N. hybridization. Arrays were then washed and stained with streptavidin-phycoerythrin, before being scanned on an Affymetrix GeneChip scanner. The probe sets contained in the Affymetrix PrimeView oligonucleotide arrays were analyzed using RMA algorithm. Hierarchical clustering was performed using Spotfire DecisionSite for Functional Genomics (Somerville).

### Treatment with epigenetic modifiers and protein inhibitors

For epigenetic modifications, cells were seeded at 50% confluence 8 hr prior to treatment with 5-Aza-2′-deoxycytidine (5-Aza 10 μM; Sigma) and valproic acid (VPA 1.5 mM; Sigma) or phenylbutyric acid (PBA 3 mM; Sigma). The drugs were continuously administered by replacing the medium every 24 h for 5 days. For pharmacological inhibition on MAP3K7, we used the commercially available inhibitor 5Z-7-oxozeaenol at a concentration of 4 mM.

### Quantitative real time PCR

#### MicroRNA

Quantification of miRNAs by *TaqMan* MiRNAs assays (Applied Biosystems) was carried out as described by the manufacturer. Briefly, 10 ng of template RNA was reverse transcribed using the *TaqMan* MicroRNA Reverse Transcription Kit and miRNA-specific stem-loop primers. Target miRNA expression was normalized between different samples based on the values of Rnu19, Rnu43 or Rnu48 expression.

#### mRNA

Total RNA was reverse transcribed using the Takara PrimeScript™ RT reagent Kit (Takara). Quantification of mRNA was carried out by *TaqMan* Gene Expression Assay. Target gene expression was normalized between different samples based on the values of Rplp0 expression.

*TaqMan* probes: MiR-377 - (000566), E2F3 Hs00605457_m1, MAP3K7 Hs01105682_m1.

For non-quantitative RT-PCR, the following primers were used:

Melan-A: F-ACTCTTACACCACGGCTGAA, R-GCTGTCCCGATGATCAAACC. Hb2M: F-TTCTGGCCTGGAGGCTATC, R-TCAGGAAATTTGACTTTCCATTC.

### Cloning and plasmids

The plasmids pMSCV-miR-377 and pMSCV-HTR were kindly provided by Agami, R [17].

- The fragments of MAP3K7, E2F3, KRAS, and CDK6 3′ UTR containing the miR-377 target site were amplified from genomic DNA (primers details in Table 1), and cloned into a psiCHECK-II plasmid (Promega) downstream of a reporter Synthetic *Renilla* luciferase gene (*hRluc*) using Xho1 and Not1 digesting. Firefly luciferase located downstream the 3′UTR fragment served as transfection internal control.
- To generate plasmid with one or more mutations in the binding site for miR-377, the seed regions were mutated from G***TGTG***AA to G***ATAT***AA, The E2F3 mRNA has 3 putative miR-377 binding sites; site-1 at 10–16, site-2 at 951–957 and site-3 at 1251–1257 of the 3′UTR, site-1 was change form, CGTGTGAA to CG***ATAT***AA, site −2 was change from TGTGTGAT to TG***ATAT***AT and site −3 was change from TGTGTGAG to TG***ATAT***AG. All mutation plasmids were generated by using Q5 Site-Directed Mutagenesis Kit (NEB).
- pGL4 containing two NF-κB DNA-binding sites linked to a luciferase cDNA sequence gene was kindly given from the Yinon Ben-Neriah lab [22].
- pGL4 containing three E2F DNA-binding sites linked to a luciferase cDNA sequence gene was kindly given from Doron Ginsberg lab [20].
- pRL-CMV Renilla used as an internal control (Promega).

**Table 1:** **Primers used to amplify the 3′UTR of the marked gene form genomic DNA**

MAP3K7	F - *CCGCTCGAG*CTCTGGGACCGTTACATTTTGA
	R- *TTTTCCTTTTGCGGCCG*CTCCAAGAATCACTGCAGGAAGA
E2F3	F - *CCGCTCGAG* CCCTGCTGCAAGAGGACTATC
	R - *TTTTCCTTTTGCGGCCGC*TGTGCCTACCATTGGGTCAG
KRAS	F – *CCGCTCGAG*TAAGGCATACTAGTACAAGTGGTAA
	R - *TTTTCCTTTTGCGGCCG*CGAACCTAAGTCACCTTCTTCCT
CDK6	F - *CCGCTCGAG* TTTCCTCCTGAGTGGCAAGC
	R – *TTTTCCTTTTGCGGCCG*CTGGAGAGAAAACAGCAGCCT

Italics letters mark the bases added to the sequences to generate sites for XhoI or NotI or PmeI.

### Luciferase assay

HEK293T or melanoma cells were seeded in 24-well plates (1 × 10^5^/well) one day before the transfection. Cells were transfected with the indicated vectors for each experiment using Polyethylenimine (PEI, Sigma). 48 h after transfection luciferase activity was determined using the dual luciferase assay system (Promega) according to the manufacturer’s instructions.

### Generation of stable melanoma cell lines

Cells were transfected with purified DNA plasmids with the Lipofectamine™ 2000 Transfection Reagent (Invitrogen) according to the manufacturer protocol. 24 h after transfection, Blasticidin antibiotic (10 μg/ml, InvivoGen) was added to the cells for selection. Following selection, the stable ectopic expression of miRNAs was repeatedly assessed using qRT-PCR.

### Determination of protein expression level by western blotting (WB)

Whole cell lysates were prepared in cell lysis buffer (50 mM Tis-Hcl, 150 mM NaCL, 0.5% sodium deoxycholate, 0.1% SDS, 1% NP-40) and supernatants collected by centrifugation. The protein concentration was measured using the Bradford assay (Bio-Rad), and 30 μg of each protein sample were denatured in SDS sample buffer and separated on 10-15% SDS–PAGE. Separated proteins were transferred to nitrocellulose membranes and the blots were blocked using 5% skim milk in PBST. Proteins were reacted with the following primary specific antibodies: GAPDH #2118, Cell-Signaling; E2F3 WH0001871M1, and MAP3K7 SAB1406506 Sigma; IκB-α (C-15) #sc-203, NFκB p65 (C-20) #sc-372 (Santa Cruz Biotechnology). Detection was carried out with horse radish peroxidase conjugated to goat anti-mouse IgG (Jackson Immuno-Research Lab), or goat anti-rabbit IgG (Sigma) followed by chemiluminescent reaction (Pierce). Densitometric analysis were done using ImageJ program.

**Nuclear/Cytoplasmic Extraction** cells was collected with 1 ml of ice-cold phosphate-buffered saline and centrifuged for 5 min at 6000 g. The supernatant was discarded, followed by another short centrifugation. The pellet was suspended in 9 volumes of HL buffer (10 mM HEPES-KOH, pH 7.5, 10 mM KCl, 3 mM MgCl2, 0.05% Nonidet P-40, 1 mM EDTA, pH 8.0) and incubated for 30 min on ice, followed by centrifugation for 5 min at 600 g, 4°C. 8 volumes of supernatant was collected into a new tube containing an equal volume of 2 × GSLB buffer (90 mM HEPES-KOH, pH 7.9, 500 mM KCl, 0.15% Nonidet P-40, 0.2 mM EGTA, pH 8.0, 20% glycerol), and defined as the “cytoplasmic fraction”. The rest of the supernatant was removed, and the pellet was washed with 9 volumes of HL buffer followed by spinning for 5 min at 600 g, 4°C. The pellet was suspended in 1 × GSLB buffer (50 mM HEPES-KOH, pH 7.9, 250 mM KCl, 0.1% Nonidet P-40, 0.1 mM EDTA, pH 8.0, 0.1 mM EGTA, pH 8.0, 10% glycerol), followed by incubation for 30 min on ice. The tubes were then centrifuged for 10 min, maximum speed, at 4°C, and the supernatant was collected into a new tube, and defined as the “nuclear fraction” [58].

### Crystal violet

Melanoma cells (5 × 10^3^) were seeded in 96-well plates and viable cell counts were monitored from seeding time (t = 0) to 96 h every 24 h. The cells were fixated with ethanol 70% and stained with crystal violet 0.1%. The color was extracted using 1% triton x-100 and absorption was read at 550 nm.

### Transwell migration

Melanoma cells (2 × 10^5^) were seeded in the upper wells of a Transwell migration system on ThinCertsTM inserts with 8-μm membranes (Greiner-bio-one) in DMEM supplemented with 0.1% FBS. The lower well contained the same medium with 10% FBS. After 24 h of incubation, the upper well content, which contained non-migrating cells, was vigorously removed using cotton swabs. The cells that migrated through the membranes were fixated with 70% cold Ethanol, stained with crystal violet 0.1% and photographed using the light microscope.

### Real-time-cell-analyzer (RTCA)

Melanoma cells were seeded in the xCELLigence DP system (Roche Diagnostics GmbH) and incubated for 1–5 days. The xCELLigence system allows for label-free and dynamic monitoring of cellular phenotypic changes in real time using impedance as readout. The system measures electrical impedance across inter-digitated micro-electrodes integrated on the bottom of tissue culture E-Plates which is then displayed as cell index (CI). Data were collected every 20 min automatically by the analyzer as described in [59]. For verification, a cellular growth curve was also obtained in parallel using the crystal violet technique described above. For monitoring migration, cells were seeded in the upper chamber in the normal culture medium of the respective cell line with 0.1% FBS. This upper chamber was then placed on the lower part of the CIM-device containing growth medium supplemented with 10% FBS as an attractant. Migration of the cells was followed for 24 h by tracking changes of the impedance signal in a CIM-plate measured on the opposing side of the membrane as described in [19].

### Soft agar assay

5 × 10^3^ cells were suspended in media containing 0.3% Select agar (Noble agar, DIFCO) and plated on a bottom layer of media containing 1% Select agar [2% agar was melted in a microwave oven, mixed 1:1 with medium (×2 DMEM medium with 20% FCS, 2% P/S mixture and 2% glutamine)] in a 96-well plate. To enable colony formation plates were incubated at 37°C for 3 weeks. Next, growth medium was removed and colonies were stained with crystal violet 0.1%. Colonies were visualized by light microscopy and were photographed at 5× magnification.

### Statistical analysis

Each experiment was performed in triplicate. Statistical significance was determined using the Student’s t-test or using two-way ANOVA. For a single comparison, a p-value < 0.05 was considered to be statistically significant.

## Additional files

Additional file 1: Figure S1. Comparison of mRNA expression in cells over expressing miR-377 to cell expressing control (A) mRNA expression profiling was performed using Affymetrix PrimeView oligonucleotide arrays according to the manufacturer’s protocol. The probe sets contained in the Affymetrix PrimeView oligonucleotide arrays were analyzed using RMA algorithm. Hierarchical clustering was performed using Spotfire DecisionSite for Functional Genomics (Somerville). The location of CDK6, MAP3K7, KRAS and E2F3 is shown. A heat-map of these probes is shown separately (B; each gene has a few probes on the CHIP array). Additional file 2:
**Data of mRNA expression array of melanoma cells mel-14PA or mel33B1 stably over-expressing either miR-377 or HTR control RNA.**
Additional file 3: Figure S2. Melan A expression in the melanoma cell lines. Total RNA was extracted form mel33B1 or mel-14PA melanoma cell, or from Skin squamous cell carcinoma (SCC). RNA was subject to RT-PCR assay using specific primers to Melan-A or hB2m.

## References

[1] Siegel R, Naishadham D, Jemal A (2013). Cancer statistics, 2013. CA Cancer J Clin.

[2] Chapman PB, Hauschild A, Robert C, Haanen JB, Ascierto P, Larkin J (2011). Improved survival with vemurafenib in melanoma with BRAF V600E mutation. N Engl J Med.

[3] Robert C, Thomas L, Bondarenko I, O’Day S, DJ M, Garbe C (2011). Ipilimumab plus dacarbazine for previously untreated metastatic melanoma. N Engl J Med.

[4] Berrocal A, Cabanas L, Espinosa E, Fernandez-de-Misa R, Martin-Algarra S, Martinez-Cedres JC (2014). Melanoma: diagnosis, staging, and treatment. Consensus group recommendations. Adv Ther.

[5] Leibowitz-Amit R, Sidi Y, Avni D (2012). Aberrations in the micro-RNA biogenesis machinery and the emerging roles of micro-RNAs in the pathogenesis of cutaneous malignant melanoma. Pigment Cell Melanoma Res.

[6] da Rocha ST, Edwards CA, Ito M, Ogata T, Ferguson-Smith AC (2008). Genomic imprinting at the mammalian Dlk1-Dio3 domain. Trends Genet.

[7] Arribas AJ, Gomez-Abad C, Sanchez-Beato M, Martinez N, Dilisio L, Casado F (2013). Splenic marginal zone lymphoma: comprehensive analysis of gene expression and miRNA profiling. Mod Pathol.

[8] Costa FF, Bischof JM, Vanin EF, Lulla RR, Wang M, Sredni ST (2011). Identification of microRNAs as potential prognostic markers in ependymoma. PLoS One.

[9] Devor EJ, DE Mik JN, Ramachandran S, Goodheart MJ, Leslie KK (2012). Global dysregulation of the chromosome 14q32 imprinted region in uterine carcinosarcoma. Exp Ther Med.

[10] Formosa A, Markert EK, Lena AM, Italiano D, Finazzi-Agro E, Levine AJ (2014). MicroRNAs, miR-154, miR-299-5p, miR-376a, miR-376c, miR-377, miR-381, miR-487b, miR-485-3p, miR-495 and miR-654-3p, mapped to the 14q32.31 locus, regulate proliferation, apoptosis, migration and invasion in metastatic prostate cancer cells. Oncogene.

[11] Gattolliat CH, Thomas L, Ciafre SA, Meurice G, Le Teuff G, Job B (2011). Expression of miR-487b and miR-410 encoded by 14q32.31 locus is a prognostic marker in neuroblastoma. Br J Cancer.

[12] Haller F, von Heydebreck A, Zhang JD, Gunawan B, Langer C, Ramadori G (2010). Localization- and mutation-dependent microRNA (miRNA) expression signatures in gastrointestinal stromal tumours (GISTs), with a cluster of co-expressed miRNAs located at 14q32.31. J Pathol.

[13] Lavon I, Zrihan D, Granit A, Einstein O, Fainstein N, Cohen MA (2010). Gliomas display a microRNA expression profile reminiscent of neural precursor cells. Neuro Oncol.

[14] Thayanithy V, Sarver AL, Kartha RV, Li L, Angstadt AY, Breen M (2012). Perturbation of 14q32 miRNAs-cMYC gene network in osteosarcoma. Bone.

[15] Zehavi L, Avraham R, Barzilai A, Bar-Ilan D, Navon R, Sidi Y (2012). Silencing of a large micro-RNA cluster on human chromosome 14q32 in melanoma: biological effects of mir-376a and mir-376c on insulin growth factor 1 receptor. Mol Cancer.

[16] Saito Y, Liang G, Egger G, Friedman JM, Chuang JC, Coetzee GA (2006). Specific activation of microRNA-127 with downregulation of the proto-oncogene BCL6 by chromatin-modifying drugs in human cancer cells. Cancer Cell.

[17] Voorhoeve PM, le Sage C, Schrier M, Gillis AJ, Stoop H, Nagel R (2006). A genetic screen implicates miRNA-372 and miRNA-373 as oncogenes in testicular germ cell tumors. Cell.

[18] Ke N, Wang X, Xu X, Abassi YA (2011). The xCELLigence system for real-time and label-free monitoring of cell viability. Methods Mol Biol.

[19] Greiner M, Kreutzer B, Jung V, Grobholz R, Hasenfus A, Stohr RF (2011). Silencing of the SEC62 gene inhibits migratory and invasive potential of various tumor cells. Int J Cancer.

[20] Schneider CA, Rasband WS, Eliceiri KW (2012). NIH Image to ImageJ: 25 years of image analysis. Nat Methods.

[21] Lindeman GJ, Gaubatz S, Livingston DM, Ginsberg D (1997). The subcellular localization of E2F-4 is cell-cycle dependent. Proc Natl Acad Sci U S A.

[22] Roh YS, Song J, Seki E (2014). TAK1 regulates hepatic cell survival and carcinogenesis. J Gastroenterol.

[23] Nabel G, Baltimore D (1987). An inducible transcription factor activates expression of human immunodeficiency virus in T cells. Nature.

[24] Fan Y, Cheng J, Vasudevan SA, Patel RH, Liang L, Xu X (2013). TAK1 inhibitor 5Z-7-oxozeaenol sensitizes neuroblastoma to chemotherapy. Apoptosis.

[25] Formosa A, Markert EK, Lena AM, Italiano D, Finazzi-Agro E, Levine AJ, et al. MicroRNAs, miR-154, miR-299-5p, miR-376a, miR-376c, miR-377, miR-381, miR-487b, miR-485-3p, miR-495 and miR-654-3p, mapped to the 14q32.31 locus, regulate proliferation, apoptosis, migration and invasion in metastatic prostate cancer cells. Oncogene. 2013. 10.1038/onc.2013.451 24166498

[26] Wang L, Shao J, Zhang X, Xu M, Zhao J. microRNA-377 suppresses the proliferation of human osteosarcoma MG-63 cells by targeting CDK6. Tumour Biol. 2015. 10.1007/s13277-014-3034-2 25577249

[27] Foster CS, Falconer A, Dodson AR, Norman AR, Dennis N, Fletcher A (2004). Transcription factor E2F3 overexpressed in prostate cancer independently predicts clinical outcome. Oncogene.

[28] Oeggerli M, Tomovska S, Schraml P, Calvano-Forte D, Schafroth S, Simon R (2004). E2F3 amplification and overexpression is associated with invasive tumor growth and rapid tumor cell proliferation in urinary bladder cancer. Oncogene.

[29] Noguchi S, Mori T, Otsuka Y, Yamada N, Yasui Y, Iwasaki J (2012). Anti-oncogenic MicroRNA-203 Induces Senescence by Targeting E2F3 Protein in Human Melanoma Cells. J Biol Chem.

[30] Dimova DK, Dyson NJ (2005). The E2F transcriptional network: old acquaintances with new faces. Oncogene.

[31] Leone G, Nuckolls F, Ishida S, Adams M, Sears R, Jakoi L (2000). Identification of a novel E2F3 product suggests a mechanism for determining specificity of repression by Rb proteins. Mol Cell Biol.

[32] Hurst CD, Tomlinson DC, Williams SV, Platt FM, Knowles MA (2008). Inactivation of the Rb pathway and overexpression of both isoforms of E2F3 are obligate events in bladder tumours with 6p22 amplification. Oncogene.

[33] Fang Y, Gu X, Li Z, Xiang J, Chen Z (2013). miR-449b inhibits the proliferation of SW1116 colon cancer stem cells through downregulation of CCND1 and E2F3 expression. Oncol Rep.

[34] Huang L, Luo J, Cai Q, Pan Q, Zeng H, Guo Z (2011). MicroRNA-125b suppresses the development of bladder cancer by targeting E2F3. Int J Cancer.

[35] Tazawa H, Tsuchiya N, Izumiya M, Nakagama H (2007). Tumor-suppressive miR-34a induces senescence-like growth arrest through modulation of the E2F pathway in human colon cancer cells. Proc Natl Acad Sci U S A.

[36] Yamaguchi K, Shirakabe K, Shibuya H, Irie K, Oishi I, Ueno N (1995). Identification of a member of the MAPKKK family as a potential mediator of TGF-beta signal transduction. Science.

[37] Schonherr M, Bhattacharya A, Kottek T, Szymczak S, Koberle M, Wickenhauser C (2014). Genomewide RNAi screen identifies protein kinase Cb and new members of mitogen-activated protein kinase pathway as regulators of melanoma cell growth and metastasis. Pigment Cell Melanoma Res.

[38] Melisi D, Xia Q, Paradiso G, Ling J, Moccia T, Carbone C (2011). Modulation of pancreatic cancer chemoresistance by inhibition of TAK1. J Natl Cancer Inst.

[39] Zhao N, Wang R, Zhou L, Zhu Y, Gong J, Zhuang SM (2014). MicroRNA-26b suppresses the NF-kappaB signaling and enhances the chemosensitivity of hepatocellular carcinoma cells by targeting TAK1 and TAB3. Mol Cancer.

[40] Kim EK, Choi EJ (1802). Pathological roles of MAPK signaling pathways in human diseases. Biochim Biophys Acta.

[41] Ray DM, Myers PH, Painter JT, Hoenerhoff MJ, Olden K, Roberts JD (2012). Inhibition of transforming growth factor-beta-activated kinase-1 blocks cancer cell adhesion, invasion, and metastasis. Br J Cancer.

[42] Martin SE, Wu ZH, Gehlhaus K, Jones TL, Zhang YW, Guha R (2011). RNAi screening identifies TAK1 as a potential target for the enhanced efficacy of topoisomerase inhibitors. Curr Cancer Drug Targets.

[43] Buglio D, Palakurthi S, Byth K, Vega F, Toader D, Saeh J (2012). Essential role of TAK1 in regulating mantle cell lymphoma survival. Blood.

[44] Liu W, Chang BL, Cramer S, Koty PP, Li T, Sun J (2007). Deletion of a small consensus region at 6q15, including the MAP3K7 gene, is significantly associated with high-grade prostate cancers. Clin Cancer Res.

[45] Wu M, Shi L, Cimic A, Romero L, Sui G, Lees CJ (2012). Suppression of Tak1 promotes prostate tumorigenesis. Cancer Res.

[46] Inokuchi S, Aoyama T, Miura K, Osterreicher CH, Kodama Y, Miyai K (2010). Disruption of TAK1 in hepatocytes causes hepatic injury, inflammation, fibrosis, and carcinogenesis. Proc Natl Acad Sci U S A.

[47] Karin M, Ben-Neriah Y (2000). Phosphorylation meets ubiquitination: the control of NF-[kappa]B activity. Annu Rev Immunol.

[48] Karin M (2006). Nuclear factor-kappaB in cancer development and progression. Nature.

[49] McNulty SE, del Rosario R, Cen D, Meyskens FL, Yang S (2004). Comparative expression of NFkappaB proteins in melanocytes of normal skin vs. benign intradermal naevus and human metastatic melanoma biopsies. Pigment Cell Res.

[50] Jackson-Bernitsas DG, Ichikawa H, Takada Y, Myers JN, Lin XL, Darnay BG (2007). Evidence that TNF-TNFR1-TRADD-TRAF2-RIP-TAK1-IKK pathway mediates constitutive NF-kappaB activation and proliferation in human head and neck squamous cell carcinoma. Oncogene.

[51] Safina A, Ren MQ, Vandette E, Bakin AV (2008). TAK1 is required for TGF-beta 1-mediated regulation of matrix metalloproteinase-9 and metastasis. Oncogene.

[52] Madonna G, Ullman CD, Gentilcore G, Palmieri G, Ascierto PA (2012). NF-kappaB as potential target in the treatment of melanoma. J Transl Med.

[53] Kashani-Sabet M, Shaikh L, Miller JR, Nosrati M, Ferreira CM, Debs RJ (2004). NF-kappa B in the vascular progression of melanoma. J Clin Oncol.

[54] Kashani-Sabet M, Liu Y, Fong S, Desprez PY, Liu S, Tu G (2002). Identification of gene function and functional pathways by systemic plasmid-based ribozyme targeting in adult mice. Proc Natl Acad Sci U S A.

[55] Lehmann F, Marchand M, Hainaut P, Pouillart P, Sastre X, Ikeda H (1995). Differences in the antigens recognized by cytolytic T cells on two successive metastases of a melanoma patient are consistent with immune selection. Eur J Immunol.

[56] Restifo NP, Marincola FM, Kawakami Y, Taubenberger J, Yannelli JR, Rosenberg SA (1996). Loss of functional beta 2-microglobulin in metastatic melanomas from five patients receiving immunotherapy. J Natl Cancer Inst.

[57] Markel G, Ortenberg R, Seidman R, Sapoznik S, Koren-Morag N, Besser MJ (2010). Systemic dysregulation of CEACAM1 in melanoma patients. Cancer Immunol Immunother.

[58] Scheinman EJ, Avni O (2009). Transcriptional regulation of GATA3 in T helper cells by the integrated activities of transcription factors downstream of the interleukin-4 receptor and T cell receptor. J Biol Chem.

[59] Solly K, Wang X, Xu X, Strulovici B, Zheng W (2004). Application of real-time cell electronic sensing (RT-CES) technology to cell-based assays. Assay Drug Dev Technol.

