# Supplementary material for: MiR-377 targets E2F3 and alters the NF-kB signaling pathway through MAP3K7 in malignant melanoma
Source: Mol Cancer. 2015 Mar 26;14:68. doi: 10.1186/s12943-015-0338-9 (PMC4392476; doi:10.1186/s12943-015-0338-9)
Supplement: Additional file 1: Figure S1. — Comparison of mRNA expression in cells over expressing miR-377 to cell expressing control (A) mRNA expression profiling was performed using Affymetrix PrimeView oligonucleotide arrays according to the manufacturer’s protocol. The probe sets contained in the Affymetrix PrimeView oligonucleotide arrays were analyzed using RMA algorithm. Hierarchical clustering was performed using Spotfire DecisionSite for Functional Genomics (Somerville). The location of CDK6, MAP3K7, KRAS and E2F3 is shown. A heat-map of these probes is shown separately (B; each gene has a few probes on the CHIP array). [file 12943_2015_338_MOESM1_ESM.pptx]

## Slide 1
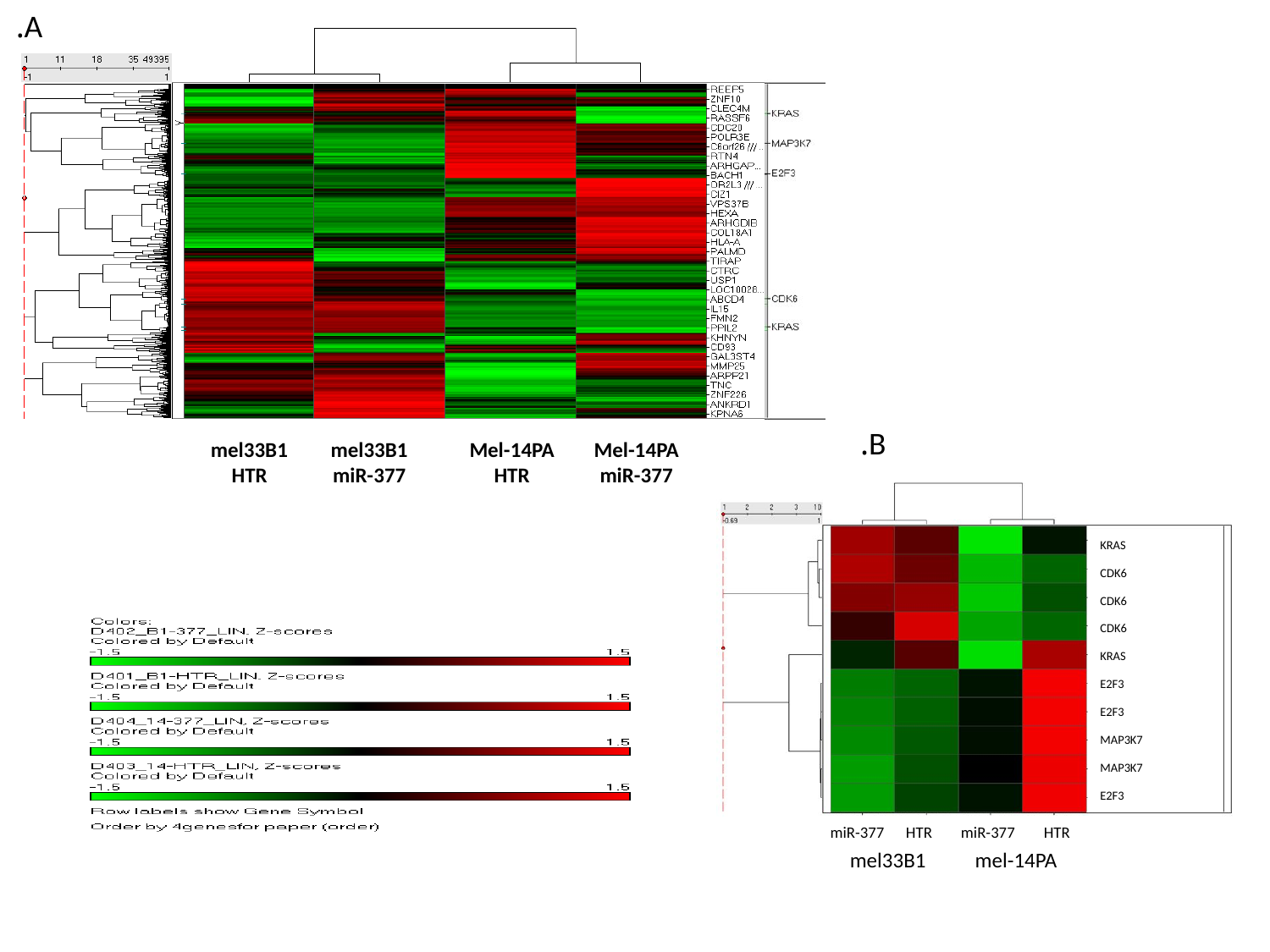

A.
mel33B1
HTR
mel33B1
miR-377
Mel-14PA
HTR
Mel-14PA
miR-377
B.
KRAS
CDK6
CDK6
CDK6
KRAS
E2F3
E2F3
MAP3K7
MAP3K7
E2F3
 miR-377 HTR miR-377 HTR
mel33B1
mel-14PA
