# Supplementary figures and images for: MiR-377 targets E2F3 and alters the NF-kB signaling pathway through MAP3K7 in malignant melanoma
Source: Mol Cancer. 2015 Mar 26;14:68. doi: 10.1186/s12943-015-0338-9 (PMC4392476; doi:10.1186/s12943-015-0338-9)

## Slide 1
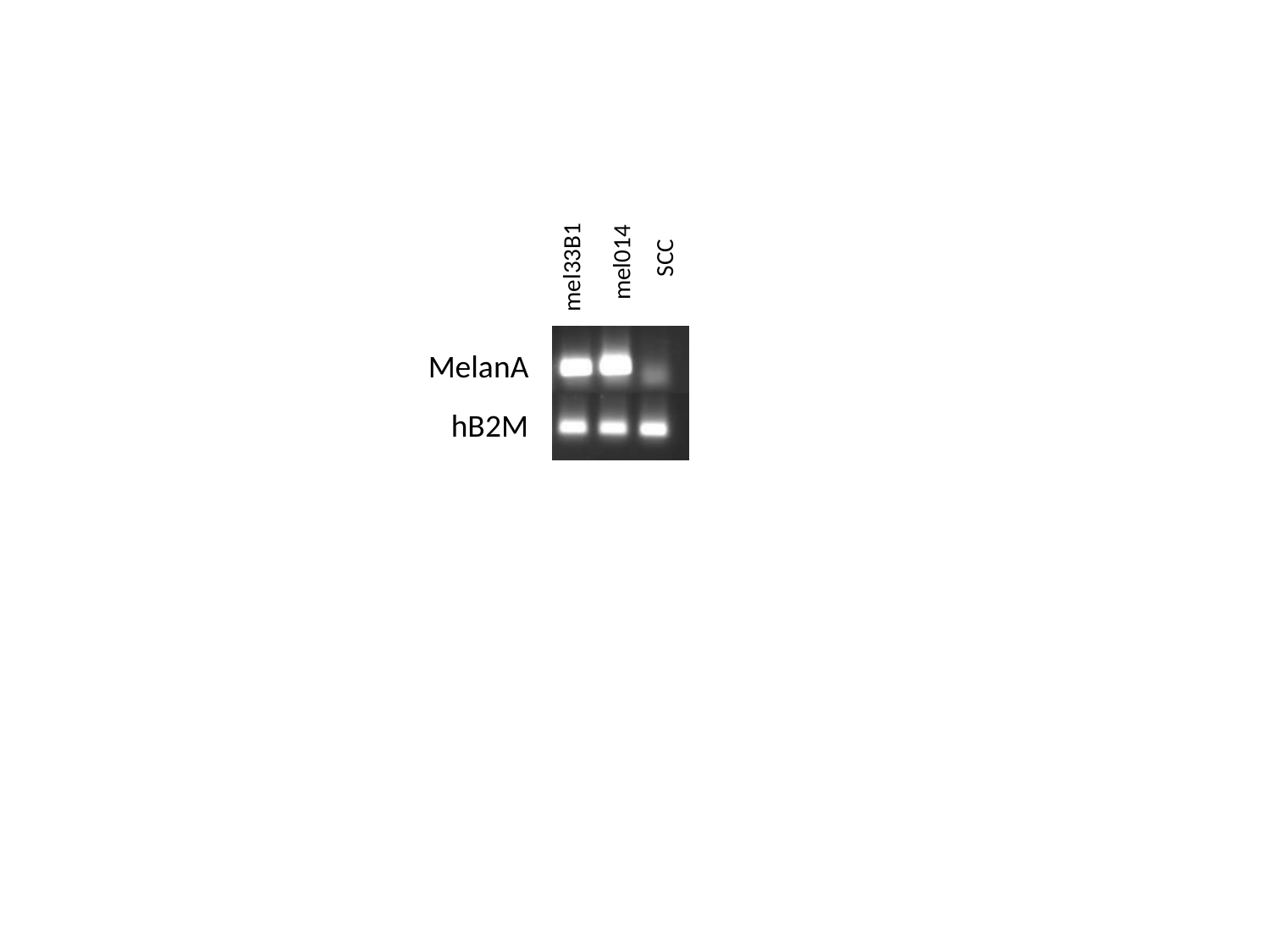

SCC
mel014
mel33B1
MelanA
hB2M

Supplement: Additional file 3: Figure S2. — Melan A expression in the melanoma cell lines. Total RNA was extracted form mel33B1 or mel-14PA melanoma cell, or from Skin squamous cell carcinoma (SCC). RNA was subject to RT-PCR assay using specific primers to Melan-A or hB2m. [file 12943_2015_338_MOESM3_ESM.pptx]
